# Characteristics of Adult Patients with Idiopathic Retroperito-Neal Fibrosis and Assessment of Risk of Relapse at Diagnosis

**DOI:** 10.3390/jcm10071380

**Published:** 2021-03-30

**Authors:** Jerome Razanamahery, Bastien Bouldoires, Sebastien Humbert, Philip Bielefeld, Veronique Fournier, Bernard Bonnotte, Gilles Blaison, Nadine Magy-Bertrand

**Affiliations:** 1 Department of Internal Medicine and Clinical Immunology, Dijon University Hospital, 21000 Dijon, France; bernard.bonnotte@chu-dijon.fr; 2 Department of Internal Medicine, Hopital Pasteur, 68000 Colmar, France; bastien.bouldoires@gmail.com (B.B.); gilles.blaison@ch-colmar.fr (G.B.); 3 Department of Internal Medicine, Besancon University Hospital, 25000 Besancon, France; shumbert@chu-besancon.fr (S.H.); nadine.magy@univ-fcomte.fr (N.M.-B.); 4 Department of Internal Medicine 2, Dijon University Hospital, 21000 Dijon, France; philip.bielefeld@chu-dijon.fr; 5 Department of Nephrology, Trevenans Hospital, 90000 Belfort, France; veronique.fournier@hnfc.fr

**Keywords:** idiopathic retroperitoneal fibrosis, risk of relapse, smoking cessation

## Abstract

Objectives: To compare adult patients’ characteristics suffering from idiopathic retroperitoneal fibrosis between “relapse-free” and relapsing patients at the diagnosis and identify factors associated with relapse at initial presentation. Methods: We conducted a retrospective multicentric study in four hospitals in Eastern France, from 1993 to 2020, of adult patients suffering from idiopathic retroperitoneal fibrosis. We analyzed clinical, biological, and radiological features at diagnosis and during a forty-month follow-up. Results: Of 47 patients suffering from retroperitoneal fibrosis, 21 patients had idiopathic retroperitoneal fibrosis. Among them, 13 experienced one or more relapses during follow-up. At diagnosis, clinical characteristics, relevant comorbidities, biological and radiological features were similar between groups. Smoking cessation seems associated with decreased relapse risk (*p*: 0.0624). A total of 8 patients developed chronic renal failure during follow-up. Ureteral infiltration at diagnosis was associated with evolution to chronic renal failure (*p*: 0.0091). Conclusion: No clinical, biological, or radiological features could predict relapse at retroperitoneal fibrosis diagnosis, but smoking cessation may prevent relapse.

## 1. Introduction

Idiopathic retroperitoneal fibrosis (IRF) is a rare inflammatory disease characterized by fibro-inflammatory tissue surrounding vessels, especially the abdominal aorta and its branches, with extension into retroperitoneal space. Fibrosis can also entrap adjacent structures such as ureters, veins, or lymph nodes [1]. Despite appropriate treatment, progression is unpredictable with a high frequency of relapse [2]. Clinical presentation is heterogeneous, from non-symptomatic patients to complications (i.e., abdominal pain, acute renal failure, lower limb edema) revealing the disease. Diagnosis is based on imaging (mostly computed tomography) showing irregular soft tissue mass surrounding the aorta and/or iliac arteries with possible extension to adjacent structures [1]. Exclusion of conditions responsible for retroperitoneal fibrosis (i.e., trauma/surgery, cancer, infections, autoimmune diseases, Ig-G4 related disease, histiocytosis, radiation therapy, and drugs) is mandatory to assess the diagnosis of IRF [3]. A biopsy is not recommended but remains useful when the mass shows atypical features suggestive of underlying conditions [1]. When undertaken, biopsy shows chronic inflammatory infiltrate and fibrosis of peri-aortic tissues, but histology is not specific [4]. Corticosteroids remain the first-line therapy with surgery to relieve obstruction of ureters in case of acute renal failure [1]. About half of patients experienced relapse or evolution to chronic renal failure during the follow-up [1,2] despite steroids. Relapse is responsible for high steroids exposure and side effects of immunosuppressive drugs. Recently, Moriconi et al. identified that smoking, lumbar pain, acute renal failure, and antinuclear antibody positivity at diagnosis were associated with relapse risk [2]. Still, few other studies have identified risk factors of relapse. From that perspective, we aimed to compare the characteristics of “relapse-free” and relapsing patients at the time of IRF diagnosis.

## 2. Materials and Methods

We performed an observational retrospective study in the Eastern France database hospital (Bourgogne-Franche Comté and Alsace) from 1993 to 2020. According to the current guidelines, all patients with an IRF diagnosis had imaging showing tissue mass surrounding vessels and increased acute-phase reactant concentrations [1]. We used standardized case report forms at diagnosis, including demographic features (age, sex), medical conditions (including smoking habits), clinical features, biochemical and hematological tests. We also collected imaging and histological features when available. Screening for Ig-G4 related disease was not routinely performed on sera of patients before 2012. Immunostaining for Ig-G4/Ig-G ratio had not been performed on tissue biopsies.

At diagnosis, acute renal failure was defined as a rapid increase of serum creatinine of more than 50% or > 88 µmol/L (1 mg/dl) when baseline serum creatinine was available. If not available, we identified acute renal failure as an increase in serum creatinine of more than 26.5 μmol/L within 48 h. A ureteral obstruction was defined by bilateral hydronephrosis on imaging. Surgical intervention was required in case of acute renal failure with no improvement of ureteral obstruction despite appropriate medical treatment. Medical and surgical interventions related to IRF were retrospectively collected, but were at the discretion of the physician. At diagnosis, radiotracer uptake of fibrosis was retained if the maximum standardized uptake value (SUVmax) on retroperitoneal fibrosis was higher than 4 on ^18^Fluorodeoxyglucose Positron Emission Tomography (^18^FDG-PET) (compared to liver reference) when performed.

Follow-up visits were at the discretion of the physician but mostly occurred at months 3, 6, 12, 24, and 36. Each patient had a complete physical examination and full biological test for C-reactive protein and serum creatinine levels. Imaging was not routinely performed during follow-up but was performed if clinical or biological findings supported suspected relapse. Progression of fibrosis was defined by the radiologist by an increase of at least 20% in the maximal transverse diameter of the mass compared with previous imaging. Relapse was defined by progressing fibrosis on imaging associated with compatible symptoms (i.e., abdominal pain, lumbar pain) and increased acute-phase reactants. Patients with isolated extending fibrosis on imaging performed for other reasons were not considered relapsing patients.

During the follow-up, chronic renal failure was defined as an estimated glomerular filtration rate < 60 mL/min/1.73 on at least two dosages separated by three months.

We tested differences between the groups using Fisher’s exact test for the qualitative data and the Student *t*-test or Mann–Whitney test for the continuous data as accurate for univariate analysis. All reported *p*-values were two-sided, and a *p*-value < 0.05 was considered statistically significant. We performed a post-hoc multivariate logistic regression analysis with backward stepwise selection to identify factors associated with relapses. All variables associated with relapse in univariate analysis with a *p*-value of less than 0.20 were included. Statistical analyses were performed using Prism 8 (GraphPad, San Diego, CA, USA).

This retrospective analysis of observational data did not require patients’ ethical approval according to standards currently applied in our country. This type of study was approved by the local ethical committee and respected the principles outlined in the Declaration of Helsinki.

## 3. Results

Forty-seven patients with retroperitoneal fibrosis were included in the study. Most of the patients were enrolled in internal medicine and nephrology departments from four hospitals (Dijon University hospital, Besancon University Hospital, Belfort, and Colmar). We excluded twenty-six patients from the study (seven for missing data, five without retroperitoneal fibrosis, five with histiocytosis, three with IgG4-related disease, three with inflammatory aneurysm, two with a malignant retroperitoneal mass, and one with radiation-induced retroperitoneal fibrosis). After exclusion, the final study group was 21 patients. Thirteen patients (61%) experienced a relapse during follow-up. At diagnosis, patients’ median age was 54 years (17–78) without difference between groups (*p*: 0.9809) with a male/female ratio of 2/1 and similar distribution between groups (*p*: 0.6251). A total of 62% of patients had a history of smoking without difference between groups (*p*: 0.9670), but former smokers tended to be more frequent in the “relapse-free” group (*p*: 0.0624). Regarding other comorbidities, hypertension, lower limb arteriopathy, and dyslipidemia were similar between groups (*p*: 0.5022; 0.332 and 0.3322, respectively), and no patients had diabetes. At diagnosis, general conditions such as fatigue, low-grade fever, and weight loss greater than 2 kg were similar between groups (*p*: 0.5720, 0.2655 and 0.3079, respectively). The most frequent clinical presentation at diagnosis was abdominal pain (57% of patients), and its frequency was similar between groups (*p*: 0.7144). Patients also presented with lumbar pain (48% of patients), which tended to be more frequent in the “relapsing” group (61% vs. 25%; *p*: 0.1140). Regarding laboratory findings, C-reactive protein (CRP) levels were similar between groups (40 mg/dL (±34) vs. 27 (±15); *p*: 0.3368). The median creatinine level was 79.8 µmol/L (48–264) at diagnosis and was similar between groups (102 µmol/L (56–264) vs. 78 µmol/L (48–193); *p*: 0.2748). Serum-positive antinuclear antibodies were a frequent condition (43%) but were similar between groups (54% vs. 25%; *p*: 0.1649) using an AAN titer at 1/80 titer; or 1/160 titer cut-off (50% vs. 16%; *p*: 0.2071). Regarding imaging, ureteral infiltration occurred in 66% of patients and did not differ between groups (*p*: 0.5489). A total of 14 patients had ^18^FDG-PET at diagnosis and before induction therapy. Radiotracer uptake on fibrosis was frequent (79 % of patients) and seemed more frequent in the “relapse-free” group (100% vs. 62.5%; *p*: 0.1044). Seven patients had a biopsy at diagnosis (five in the relapse group vs. two in the relapse-free group). Histological samples were similar with non-specific infiltrate and fibrosis (*n* = 7). The pathologist did not perform a search for Ig-G4 related disease on immunostaining on biopsy samples. Regarding treatment, almost all patients (90%) had corticosteroids at diagnosis. In the “relapsing“ group, 11/13 patients received a corticosteroid regimen at 1 mg/kg/day (one patient received 0.7 mg/kg/day, and one received no treatment). In the “relapse-free” group, 5/7 received a corticosteroid regimen of 1 mg/kg/day (one received 0.5 mg/kg/day and the data for one were missing). Nearly half (43%) of the patients underwent ureteral stenting by double-J catheters, but the proportion was similar between groups (31% vs. 62.5%; *p*: 0.1694).

All characteristics of patients at diagnosis are reported in Table 1 and Table 2.

The median duration of follow-up was 40 months (14–264). During follow-up, 13 patients experienced at least one relapse. The median duration between diagnosis of IRF and relapse was 12 months (3–72). The median relapse rate per patient was 1 (1–3). Patients with relapse received azathioprine (*n* = 5), methotrexate (*n* = 5), mycophenolate mofetil (*n*= 4), and tamoxifen (*n* = 1). The main reasons for treatment changes were lack of efficacy or non-tolerable side effects. A total of three patients underwent ureteral stenting by double-J catheters during follow-up. CRP and creatinine levels were similar at months 3, 6,12, 24, and 36, but the mean CRP level was higher at 36 months in patients with relapse (3.9 ± 1.66 vs. 1.8 ± 0.9; *p*: 0.0411). At the end of follow-up, eight patients (five in the “relapse” group and three in the “relapse-free” group) suffered from chronic renal failure (estimated glomerular filtration rate < 60 mL/min/1.73 on at least two dosages separated by three months). The only factor associated with chronic renal failure progression was the ureteral infiltration at baseline (100 % vs. 46 %; *p*: 0.0091). Creatinine levels at month 3 were more elevated in the non-chronic renal failure group and were approaching statistical significance (79.51 ± 13.80 vs. 108.5± 35.9; *p*: 0.0583). Relapse was not associated with evolution to chronic renal failure. No factors (former smokers, lumbar pain, positive ANA titers, or radiotracer uptake on fibrosis in ^18^FDG-PET) were correlated with an absence of relapse on multivariate analysis.

## 4. Discussion

We herein reported on a multicentric study comparing patients’ phenotypes at IRF diagnosis and establishing factors associated with relapse. Our data suggest smoking cessation seems related to the absence of relapse, but no features (clinical, biological, or imaging) could predict relapse at diagnosis of IRF. The frequency of relapse in our cohort was similar to that reported in the literature [2,5,6] (Table 3). Demographic features confirmed middle-aged males’ predominance, but the male/female ratio in the current study is one of the lowest reported in the literature. Our data suggest that lumbar pain might also be associated with relapse. Moriconi et al. established that lumbar pain was statistically associated with relapse risk [2]. Based on those two observations, we encourage physicians to perform early imaging to shorten delays in diagnosing IRF, especially in middle-aged men with increased CRP levels. Disease activity is difficult to assess with only biological tests and conventional imaging (CT or MRI). Thus, evaluation of activity with ^18^FDG-PET was proposed by analogy with other inflammatory diseases. Still, the correlation between metabolic activity and elevated acute-phase reactants is not perfect [7,8], as uptake on fibrosis can remain present despite the normalization of acute-phase reactants [7]. This imperfect correlation could bias the interpretation of relapse. Few studies report interest in early metabolic evaluation at 6 months to predict relapses [8], and no correlation has been established between metabolic activity and risk of relapse at diagnosis, which is consistent with our results. Unfortunately, we could not provide a detailed radiological description of the patients at diagnosis and during follow-up. However, we believe that radiological features (such as length, thickness, and density of fibrosis) could be of interest during early follow-up to identify non-responders; and propose early immunosuppressive agent therapies irrespective of acute-phase reactants. To our knowledge, only one study established the efficiency of contrast enhancement and diffusion coefficient to differentiate active from chronic lesions on MRI [9]. Those evaluations could be correlated with metabolic activity on ^18^FDG-PET for the staging of the disease. There is little data about progression to chronic renal disease in IRF; it has been assessed to be between 24% [2] and 42% [4], consistent with our observed prevalence of 37%. In the current study, ureteral infiltration was associated with the evolution to chronic renal failure. Patients presenting with this radiological feature must have a complete renal examination at each visit to identify early renal damage. Of interest, a high creatinine level at three months seems to be protective from chronic renal failure. But this result is probably related to higher creatinine levels in relapse-free patients at baseline. Nevertheless, this surprising finding needs to be evaluated by other studies.

The pathophysiology of IRF is complex. Infiltration of inflammatory cells in the aortic wall could result from low-density lipoprotein oxidation with ceroid formation in atherosclerotic plaques, presumably by advanced lesions with inflammation/fibrosis extending to the adventitia and beyond [12]. Those endogenous agents are responsible for TGF-β production and interleukine-4, T, and B-cell activation secondary to complex immune tracking [1,13]. Interestingly, no TNF-alpha was identified in patients’ aortas, suggesting that TNF-alpha is not a significant factor in retroperitoneal fibrosis development [11]. Thus, TNF-alpha inhibitors must not be considered as efficacious therapy in IRF [11]. Nicastro et al. have recently established the role of Th2 polarization in fibrocyte recruitment [14].

Our study suggests a protective role for smoking cessation in relapse of IRF. Cigarette exposure reduces NF-kB, JAK/STAT, and MAP-kinase pathway signaling leading to M2 and M2-like polarization of macrophages [15,16]. The M2 pathway promotes Th2 polarization by CCL17 and CCL2 and IL-4 production by basophils, and promotes tissue remodeling via TGF-β produced by Th2 lymphocytes [17]. The M2-like pathway interacts with B-cell lymphocytes via IL-10 [17]. Interestingly, TNF-alpha production is promoted by M1 polarization, which is not overexpressed by smoking exposure. We hypothesize that cigarette exposure can interplay with macrophage polarization and ultimately promote fibrosis. Studies of specific surface expression cytokine and chemokines on retroperitoneal biopsy tissue samples of smokers and former smokers could be of interest to confirm this hypothesis.

Another study supports pseudo-vasculitis of vasa vasorum in retroperitoneum small vessels promoting media thickening and pseudoaneurysm [18]. The extension of inflammation induced by necrotizing vasculitis can extend into the retroperitoneal space with immune-mediated consequences, as reported earlier [1,13]. To date, an initial trigger of vasculitis has not been identified. Recently, Ig-G4 related disease was identified as a mimicker of IRF [19] but was distinguished from IRF despite similar pathophysiology (TGF-β promotes fibrosis, interleukin-5 implication via Th2 lymphocytes, and B-cell activation). As Rituximab (Cd-20 antibody receptor) has shown consistent and reliable results in the treatment of Ig-G4 related disease [20], it is a promising therapeutic option to consider as second-line therapy.

There are some limitations to our study, mostly caused by study design. First of all, it is a retrospective study with data collection based on patient records. At diagnosis, searches for Ig-G4 related disease were not routinely performed, especially for the oldest diagnoses. As Ig-G4 related disease has similarities (presentation, response to steroids) with IRF, some IRF could be Ig-G4 related disease according to the current guidelines for this entity [19]. The small sample size may reduce the confidence of statistical analyses. Additionally, the non-standardized surveillance could bias the relapse rate, especially in non-symptomatic patients. However, as reported in the literature, IRF is a rare condition, especially in Eastern countries compared to such as China, suggesting a racial difference in IRF’s occurrence.

Nevertheless, our study’s major strength is its data derivation from four centers, including two tertiary centers, covering 27 years of long-term follow-up data.

To conclude, relapse is a frequent situation in IRF, and no factor is associated with the risk of relapse at diagnosis, but smoking cessation may prevent relapse.

## Figures and Tables

**Table 1:** Characteristic of patients at diagnosis.

	Total (*n* = 21)	Relapse (*n* = 13)	Relapse Free (*n* = 8)	*p* Value
Mean age (+ /-SD)	53.90 ± 13.78	53.84 ± 11.51	54 ± 17.76	0.9809
Median age	54 (17–78)	50 (39–78)	58 (17–72)	0.9809
Sex M (%)	12 (57)	8(61)	4 (50)	0.6251
Weight	72.6 (±16.06)	72.6 (±16.36)	72.6 (±17.55)	>0.9999
Relevant comorbidity at baseline				
History of smoking *n* (%)	13 (62)	8 (61)	5 (62.5)	0.9670
Current smoker *n* (%)	11 (52)	8 (61)	3 (38)	0.3079
Former smoker *n* (%)	2 (10)	0 (0)	2 (25)	0.0624
Never smoker *n* (%)	8 (38)	5 (38)	3 (38)	0.9670
High blood pressure *n* (%)	6 (29)	3 (23)	3 (38)	0.5022
Diabetes mellitus	0 (0)	0 (0)	0 (0)	not possible
Lower limb arteriopathy *n* (%)	3 (14)	1 (8)	2 (25)	0.3322
Dyslipidemia *n* (%)	3 (14)	1 (8)	2 (25)	0.3322
Clinical characteristics at baseline				
Fatigue	4 (19)	3 (23)	1 (12.5)	0.5720
Low-grade fever	2 (10)	2 (15)	0 (0)	0.2655
Weight loss > 2% BMI	11 (52)	8 (61)	3 (38)	0.3079
Lumbar pain	10 (48)	8 (61)	2 (25)	0.1140
Abdominal pain	12 (57)	7 (54)	5 (62.5)	0.7144

**Table 2:** Laboratory examination, radiological features and treatments at baseline.

	Total (*n* = 21)	Relapse (*n* = 13)	Relapse Free (*n* = 8)	*p* Value
Laboratory tests at diagnosis				
Mean C Reactive Protein	35 ± 28	40 ± 34	27 ± 15	0.3368
Median C Reactive Protein	27 (3.6–131)	17 (1–131)	26 (3.6–49.7)	
Mean Hb	12.5 ± 2.4	12.8 ± 2.40	12.1 ± 1.7	0.4922
Median Hb	12 (9.2–15.9)	12.7 (9.2–15.9)	11.8 (10.3–15.4)	
Mean creatinine level	103 ± 55	91 ± 42	120 ± 72	0.2748
Median creatinine level	79.8 (48–264)	78 (48-193)	102 (56–264)	
Mean creatinine clearance	79 ± 32	86 ± 28	68 ± 35	0.2578
Median creatinine clearance	84 (16–123)	84 (37–123)	54 (16–117)	
Positive antinuclear antibodies (>1/80)	9 (43)	7 (54)	2 (25)	0.1649
Positive ANCA antibodies	0 (0)	0 (0)	0 (0)	not possible
Radiological findings at diagnosis				
Ultrasound for diagnosis *n* (%)	5 (24)	2 (15)	3 (38)	0.3739
Computed tomography *n* (%)	21 (100)	13 (100)	8 (100)	not possible
Periaortic infiltration *n* (%)	21 (100)	14 (100)	9 (100)	not possible
Ureteral infiltration *n* (%)	14 (66)	8 (61)	6 (75)	0.5489
FDG-PET at diagnosis *n* (%)	14 (66)	8 (61)	6 (75)	0.5490
PET findings at diagnosis				
Radiotracer uptake on fibrosis *n* (%)	11 (79)	5 (62.5)	6 (100)	0.1044
No radiotracer uptake on fibrosis *n* (%)	3 (21)	3 (37.5)	0 (0)	0.1044
Biopsy at diagnosis *n* (%)	6 (29)	5 (62.5)	1 (12.5)	0.2205
Surgical interventions				
Double-J catheters *n* (%)	9 (43)	4 (31)	5 (62.5)	0.1694
Ureterolysis *n* (%)	1 (4.8)	1 (8)	0 (0)	0.4489
Medical treatment at diagnosis				
Corticosteroids *n* (%)	19 (90)	12 (92)	7 (87.5)	0.7318
Immunosuppressive agents *n* (%)	0 (0)	0 (0)	0 (0)	not possible
Mean steroids duration	26 ± 13	30 ± 15	23 ± 10	0.4383
Median steroids duration	26 (12–48)	36 (12–48)	20 (14–36)	0.4383

**Table 3:** Description of retroperitoneal fibrosis reported in literature.

Authors	Current Study	Gallais Sérézal [10]	van Bommel [5]	Vaglio [11]		Fernandez-Codina [4]	Scheel [3]	Marcolongo [6]	Moriconi [2]
Study design	Multicentric retrospective	Multicentric retrospective	Monocentric prospective	Randomized control trial		Monocentric retrospective	Monocentric retrospective	Monocentric retrospective	Monocentric retrospective
Country	France	France	Netherlands	Italy		Spain	USA	Italy	Italy
Years duration	1993–2020	1987–2011	1998–2008	2000–2006		1982–2009	2003–2009	1990–2002	2004–2018
Number of patients	21	30	53	18 (steroids)	18 (tamoxifen)	24	48	26	43
Age at diagnosis	53.90 ± 13.78	55 ± 13	64	56	61	51 ± 16	54,2	56	55
M/F ratio	1.33/1	4.9/1	3.3/1	2/1	1.5/1	3.8/1	1.18/1	2.7/1	1.6/1
Duration of symptoms before diagnosis (month)	NA	1.2 ± 10	8.3	NA	NA	5.2 ± 5.1	NA	NA	NA
Pain (%)	12 (57)	25 (83)	49 (92)	16 (89)	17 (94)	19 (79)	45 (94)	NA	20 (47.5)
Tobacco (%)	13(62%)	20 (67)	22 (41)	ND	ND	NA	NA	NA	18 (48)
CRP value at diagnosis (mg/L)	35 ± 28	45 ± 36	23	26.5	27.5	NA	NA	NA	22.5
Creatinine level at diagnosis (µmol/L)	103 ± 55	156 ± 151	124	137	128	492 ± 413	NA	NA	NA
Number of biopsies (%)	7(33)	11 (37)	NA	10 (56)	10 (56)	13 (54)	NA	5 (19)	20 (0.46)
Duration of follow-up (months)	40	65 ± 65	NA	57	54	24	NA	49	90 ± 56
Relapse	13 (61)	20 (69)	ND	3 (17)	9 (50)	1 (4)	ND	7 (27)	15 (34)
Chronic renal failure	8 (38)	7 (24)	ND	ND	ND	10 (42)	ND	ND	13 (32)

## Data Availability

Data available in a publicly accessible repository.
